# Context-dependent memory effects in two immersive virtual reality environments: On Mars and underwater

**DOI:** 10.3758/s13423-020-01835-3

**Published:** 2020-11-17

**Authors:** Yeon Soon Shin, Rolando Masís-Obando, Neggin Keshavarzian, Riya Dáve, Kenneth A. Norman

**Affiliations:** 1 Princeton Neuroscience Institute, Princeton, NJ USA; 2 Department of Psychology, Princeton University, Princeton, NJ USA

**Keywords:** Context-dependent memory effect

## Abstract

The context-dependent memory effect, in which memory for an item is better when the retrieval context matches the original learning context, has proved to be difficult to reproduce in a laboratory setting. In an effort to identify a set of features that generate a robust context-dependent memory effect, we developed a paradigm in virtual reality using two semantically distinct virtual contexts: underwater and Mars environments, each with a separate body of knowledge (schema) associated with it. We show that items are better recalled when retrieved in the same context as the study context; we also show that the size of the effect is larger for items deemed context-relevant at encoding, suggesting that context-dependent memory effects may depend on items being integrated into an active schema.

## Introduction

Returning to an alma mater for a reunion can bring back memories from the past. Walking by campus may make it easier to recall past events that took place in the dorms, classrooms, and dining halls, even when those memories are not easily retrievable elsewhere. This flood of memories when returning to an old environment is known as the environmental reinstatement effect (Smith, 1979). Research in episodic memory explains this effect with the encoding-specificity hypothesis, which posits that increasing levels of overlap with the encoding context during retrieval aids memory performance (Tulving & Thomson, 1973). However, the beneficial effect of context reinstatement in recall has not been consistently supported in the memory literature (for a review, see Smith & Vela, 2001). Contexts have been manipulated in various ways such as background colors (Isarida & Isarida, 2007; Weiss & Margolius, 1954) and physical rooms (Eich, 1985; Fernandez & Glenberg, 1985), but these manipulations do not always lead to a context-reinstatement effect (Fernandez & Glenberg, 1985; Isarida & Isarida, 2007; Wälti et al., 2019). The discrepancy between the strong anecdotal psychological experience and weak experimental evidence indicates that extant experimental paradigms are missing key features that are responsible for evoking context-dependent memory in real life. What, then, are these missing features?

One of the seminal studies that showed strong context-dependency used rich, real-world environments to manipulate the congruency between encoding and retrieval contexts (Godden & Baddeley, 1975). They found that scuba divers recalled learned words better when the retrieval context (underwater or land) matched the encoding context. It is worth noting, however, that subjects were put into vastly different situations that likely activated different bodies of knowledge associated with each environment (e.g., how to swim and breathe underwater). Similarly, Smith and Manzano (2010) showed a robust context reinstatement effect in a study where contexts were manipulated by video scenes showing situations that subjects were likely to be already familiar with. Relatedly, a change in the mental representation of the current situation can reduce access to episodes that happened before the change (for a review, see DuBrow et al., 2017). These studies suggest that, in order to demonstrate a robust context effect, subjects should mentally represent distinct situations and activate distinct sets of knowledge while encoding and retrieving the items. Standard laboratory experiments that merely change the physical environment or the color on a screen may have failed to elicit the effect because they failed to make subjects believe that they were in different situations.

These ideas fit with prior work in cognitive psychology (Alba & Hasher, 1983; Bransford & Johnson, 1972) and cognitive neuroscience (Gilboa & Marlatte, 2017; Poppenk & Norman, 2012; Preston & Eichenbaum, 2013; Schlichting & Preston, 2015; Tse et al., 2007; van Kesteren et al., 2012; Whittington et al., 2019) showing that activation of relevant pre-existing knowledge (i.e., schemas) can facilitate new learning by providing a “scaffold” onto which new information can be attached. Once information has been attached to this contextual scaffold, reinstating the scaffold at test should facilitate recall, and taking it away should hurt recall, thereby leading to a context change effect.

**Fig. 1:**
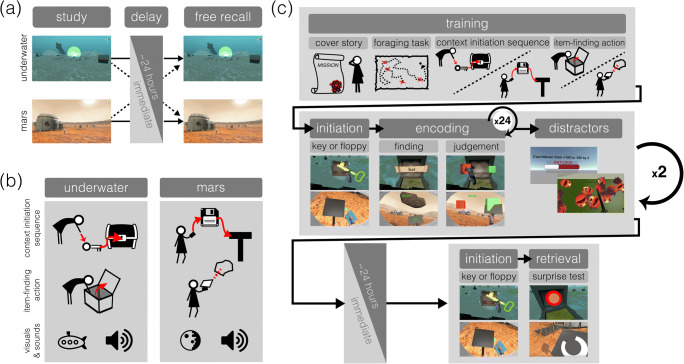
Methods pipeline. (**a**) Study design. We used a 2 (study-test context congruency: same vs. different) × 2 (study-test interval: immediate vs. delay) between-subjects design. Subjects were split between getting tested in either the same environment where learning occurred or in the other environment. Dotted arrows indicate the "different" condition while solid arrows indicate the "same" condition. Subjects went into the free-recall session either immediately or approximately 24 h after learning of the items occurred. (**b**) Environment-specific gameplay mechanics and stimuli. The Underwater (UW) and Mars planet (MP) environments each contained context-specific gameplay mechanics (top and middle rows), with distinct visual and auditory stimuli (bottom row). (**c**) Task procedure. Our paradigm consisted of three main phases (delineated by the three rows). Subjects first read the cover story and were familiarized with each of the environments (top row) through the following tasks: (1) a foraging task, (2) the context-specific initiation sequence, and (3) item-finding actions. Each practice run was followed by the two distractor tasks. In the second phase (middle row), each subject went through two encoding sessions in one of the two environments. Before encoding, subjects performed the context-initiation sequence specific to the environment. They then found to-be-remembered items by performing the environment-unique item-finding action and judged whether the item was useful or not for survival in the present environment. They did this for 24 items in a given session before proceeding to two distractor tasks (right-most middle row). The encoding session was performed twice in the same environment with two non-overlapping sets of word items. Either immediately following the encoding session or after a 1-day delay (bottom row), subjects again performed the context-initiation sequence specific to the retrieval environment, at which point they were asked to recall all the words they had judged during study (i.e., 48 words)

In the present study, we aimed to develop an experimental paradigm that can produce a strong context reinstatement effect in a laboratory setting. First, we used two virtual reality (VR) environments that we predicted would activate distinctive pre-existing sets of knowledge (i.e., schemas) in the subjects, underwater (UW) and Mars planet (MP) environments. In other words, these two environments differed not only perceptually, but also in their prior conceptual associations. Furthermore, to maximize the difference between the two contexts while expanding on their existing knowledge, we also had subjects perform context-specific actions that were physically distinctive: subjects performed downward motions in UW and upward motions in MP when interacting with objects to initiate each session (i.e., context-initiation action sequences) and to discover to-be-remembered items (i.e., item-finding actions; Fig. 1b). Second, in order for subjects to have these bodies of knowledge readily available at the time of study and test, as was the case for the divers in Godden and Baddeley (1975), we familiarized subjects with the virtual environments in a foraging task where they collected objects dispersed throughout each environment. This was followed by a practice of the associated sequences of actions (context-initiation action sequences and item-finding actions) before they went into the main task (Fig. 1c, top row).

Another key desideratum is that, during encoding, these distinct bodies of knowledge should be activated when performing the experimental tasks. If subjects are aware of a future memory test, they may use mnemonic strategies unrelated to the present context and ignore other inputs from the environment. Thus, to ensure contextual relevance during encoding and to conceal the purpose of the study, we used a surprise memory test. Furthermore, a cover task for encoding forced subjects to deliberately integrate the memory items into the given context (Eich, 1985). Specifically, we asked subjects to judge whether a shown item was useful in the context where it was found. We hoped that these judgment tasks that were unique to each environment, paired with the item-finding and context-initiation action sequences (also unique to each environment), would foster the activation of context-unique associations during encoding. Additionally, we hypothesized that items that were affirmatively judged to be useful in the encoding environment would show a stronger context-change effect – the idea being that schema-consistent items would be more strongly integrated into the active schema at encoding (Bransford & Johnson, 1972; Spalding et al., 2015; van Kesteren et al., 2013, 2018, 2020) and thus suffer more if that schema were not active at retrieval. The context-initiation action sequences were also performed at the beginning of the retrieval session, making the encoding and retrieval sessions more similar for the “same” condition and more distinctive for the “different” condition.

Lastly, we also manipulated the interval between encoding and retrieval. When items were encountered very recently, they may be retrievable without relying on contextual cues, and this may weaken context dependency (Smith & Vela, 2001). To test this, we used two levels of encoding-retrieval intervals, where a longer interval (the “delay” condition) was expected to produce a stronger reinstatement effect than a shorter interval (the “immediate” condition).

In summary, our approach was to combine as many factors as we could in the service of ensuring that participants activated distinct bodies of knowledge (i.e., schemas) when learning word lists in the two contexts. The benefit of this combined approach is to maximize effect size (if our hypothesis is correct), with the complementary drawback that – if we obtain an effect – we are not in a position to say which of the factors are necessary and sufficient for driving the effect.

## Methods

### Participants

Seventy-two adults (50 female; 22 male) recruited from Princeton University and the university community participated in our study. All but two participants were right-handed (one left-handed, one ambidextrous). Informed consent was obtained from all participants in accordance with Princeton Institutional Review Board, and subjects were each provided with monetary compensation or course credit for participating in the study. With the exception of eight participants who did not complete the study due to technical issues with the VR devices and/or Unity (e.g., VIVE wireless disconnected or screen froze during encoding), a total of 64 subjects were included in the analyses. We decided the sample size by bootstrapping from our pilot data and choosing a number of subjects who satisfied all necessary counterbalancing constraints.

### Task and procedure

We used a 2 (study-test context congruency: same vs. different) × 2 (study-test interval: immediate vs. delay) between-subjects design (Fig. 1a). The experimental procedure was divided into three parts: a training phase with sessions in both UW and MP (Fig. 1c, top row), two encoding sessions in one of the two environments (Fig. 1c, middle row), and a retrieval session either in the original encoding environment (the “same” condition) or in the other environment (the “different” condition; Fig. 1c, bottom row). In the “immediate” condition, encoding and retrieval occurred on the same day, while for the “delay” condition, retrieval took place the day after encoding.

#### Training

The training session is depicted in Fig. 1c, top row. After signing consent and screening forms, subjects were handed a paper with a fictionalized mission statement outlining the task to be performed in VR. Subjects were told that they were pioneers developing alternative places for humans to inhabit in the future and instructed to judge whether they should keep the items they discover based on the usefulness and pertinence to thriving in the environment they were in. This was meant to obscure the purpose of the study and the surprise memory test later.

After reading the mission statement, subjects were familiarized with the VR software by entering a practice environment. In this environment, subjects learned how to navigate and interact with objects that they would encounter in the main task. Subjects learned how to navigate by teleportation and how to find and judge items for the cover task (e.g., bending down, reaching upwards, etc.). Experimenters communicated with subjects during training via an intercom system connected to the head-mounted display audio-device.

Subjects were then introduced to the MP and UW environments. The order of the environments was counterbalanced across subjects. In each environment, subjects first performed a foraging task where subjects explored and collected 20 floating spheres scattered across the environment. This was followed by a context-initiation action sequence (Fig. 1b, top row), which subjects were required to perform at the beginning of each session. For this, subjects in UW needed to bend down to reach a key and insert it into the key-hole of a large treasure chest in the center of the environment, while subjects in MP needed to reach upwards to grab a floppy-disk and insert it to the side of a podium near the center of the crater. After the context-initiation action sequence, subjects heard an audio instruction for the task they needed to perform in the session. The instruction guided subjects to practice item-finding actions and the judgment mechanics that required reaching out and picking either a red or green cube with their controller. The item-finding actions were also unique to the environment. Subjects in UW found a to-be-remembered item by bending down and digging inside of a chest, whereas MP subjects did so by reaching up to a floating rock and scanning it while it was latched onto the scanner (Fig. 1b, middle row). After finding and judging four items in the environment, they were teleported out and asked to perform two types of distractor tasks. The first distractor task was a countdown task in which subjects were instructed to count down from 100 to negative 300 by a randomly-generated single-digit number in an empty environment. The second distractor task was a monster-smash task that required subjects to hit as many monster heads off the ground as possible. The countdown task and monster-smash task both lasted 1 min and were always performed in succession via virtual transportation from the countdown room to the monster smashing platform. Subjects repeated these tasks when trained in the second environment.

#### Encoding

After the training phase was completed, subjects were transported to either MP or UW for the encoding sessions. There were two encoding sessions (Fig. 1c, middle row). At the beginning of the encoding session, subjects first performed the context-initiation task that was specific to the environment, after which they listened to the recorded instruction. Subjects then performed a cover task where they made judgments about whether the items they discovered should be kept in the environment based on their perceived usefulness for surviving in that environment. There were 24 items in each session, and they were told that they should only keep roughly half of the items. Immediately after the word disappeared, the judgment cubes (green for useful, red for harmful) appeared in front of the subjects’ virtual visual field simultaneously and remained there for 6 s before disappearing. If no selection was made within the 6-s trial window, the judgment trial was counted as missed.

After successfully discovering all 24 items in chests (in UW) or rocks (in MP), subjects performed distractor tasks (i.e., countdown and monster-smash tasks). Once both distractors were completed, subjects were virtually transported back to the same encoding environment as the first encoding session and initiated the second encoding session. The second encoding session was identical to the first except for the to-be-remembered items (24 new words). After the second encoding session, subjects again performed the two distractor tasks.

#### Retrieval

Following the encoding task, subjects were introduced to the retrieval session (Fig. 1c, bottom row). Subjects in the "immediate" condition continued to the retrieval task while subjects in the "delay" condition were told to return the next day for additional missions. Before the retrieval session, subjects in the "delay" condition were re-familiarized with the retrieval environment and performed the two distractor tasks. Subjects in the "immediate" condition were transported to the retrieval environment immediately after the distractor tasks that followed the second encoding session.

The “same” condition subjects returned to the same environment in which encoding took place for retrieval (i.e., UW-UW or MP-MP), while the “different” condition subjects faced the surprise memory test in the environment that differed from the encoding session (i.e., UW-MP or MP-UW).

Once subjects were virtually transported to the retrieval environment, subjects performed the corresponding context-initiation task after which they learned for the first time that there was a surprise memory test. Subjects were instructed to verbally recall all the words they had discovered from both encoding sessions (i.e., 48 words), regardless of whether words were judged to be useful or not. The recall period lasted 2 min.

### Materials

#### Environments

Two distinctive VR environments were custom-built and explored by subjects with a wireless head-mounted virtual reality display (HMD): underwater (UW) and Mars planet (MP). To maximize the difference between the two contexts, each had a different layout and a set of thematically corresponding specific player actions, objects, and sounds (Fig. 1b). Sound effects were obtained online or made in-house with Ableton Live software and instruction audios were created using a text-to-speech service (fromtexttospeech.com).

The UW environment resembled the ocean floor. The landscape was tinted blue with coral surrounding the play area, and 3-D models of shipwrecks, submarines, boats, and chests were scattered around the environment. The words were located inside small chests; to interact with the chests, subjects had to bend down, lift the lid, and place their controller inside the chest to “dig” into the chest to reveal the word to judge. In addition to the bubble and underwater sounds, all instructions in the UW context were given by a male voice.

The MP environment resembled science fiction depictions of the planet Mars. The landscape was tinted orange with mountains far in the distance of a large crater, which served as the play area. Models of spaceships, satellites, and floating rocks were interspersed around the crater with additional spaceships hovering in the sky. In MP, to interact with the floating rocks, subjects had to lift the rocks with their controller and press the trigger button to “scan” them and reveal the word. To maximize immersion, coarse wind sounds played in the background, and static sound effects were triggered with every word discovery (e.g., “scanning rock”). All instructions in the MP context were given by a female voice.

#### Words

Forty-eight concrete noun words were used as encoding items. The same set of 48 words was presented for both MP and UW. To ensure that roughly half of the items were judged useful in both environments, we normed the words using Mechanical Turk, where the context was given by a screenshot of the corresponding VR environment and 180 words were judged for usefulness in the given environment. The mean probability of the chosen set of words being judged useful was 0*.*56 (*SD* = 0*.*28) in MP and 0*.*48 (*SD* = 0*.*25) in UW.

### Apparatus

All tasks were presented on a wireless HTC Vive Pro head-mounted display (1,440 *×* 1,600 resolution per eye, with a 90-Hz refresh rate, and built-in headphones and integrated microphone), which was wirelessly connected (with a HTC Wireless Adapter) to a custom-built computer running 64-bit Windows 10 on an Intel Core i7-7800X CPU @ 3.50GHz with 32GB RAM and an Nvidia GeForce RTX 2080 graphics card.

All tasks were created and coded in Unity3D 2017.4.3, a game-development platform, with Virtual Reality Toolkit (VRTK; vrtk.com), a virtual-reality programming tool-kit for Unity3D. 3D models, textures, environments and other assets were downloaded from the Unity Asset Store (assetstore.unity.com) and Turbosquid (turbosquid.com) and then modified or custom-built using Blender (blender.org).

### Statistical analyses

We performed statistical analyses using a generalized linear mixed-effects model in R with the “lme4” package (Bates et al., 2015), treating subjects and word stimuli as random effects, and treating context congruency, encoding-retrieval interval, and usefulness as fixed effects. For confidence intervals, we performed bootstrapping, sampling different sets of subjects with replacement 5,000 times.

## Results

### Encoding

Overall, subjects spent 7.44 mins per encoding session (*SD* = 0*.*92 min, 95% bootstrap CI [7*.*23, 7*.*68]). Subjects spent more time in MP (*M* = 7*.*89 min, *SD* = 0*.*99 min, 95% bootstrap CI [7*.*57, 8*.*25]) than in UW (*M* = 7*.*00 min, *SD* = 0*.*58 min, 95% bootstrap CI [6*.*80, 7*.*20]; *t*(49*.*83) = 4*.*380, *p <* 0*.*001), reflecting longer distance (in arbitrary units) traveled in MP (*M* = 6*.*13, *SD* = 0*.*49, 95% bootstrap CI [5*.*97, 6*.*31]) than in UW (*M* = 5*.*00, *SD* = 0*.*55, 95% bootstrap CI [4*.*81, 5*.*19]); *t*(61*.*29) = 8*.*714, *p <* 0*.*001).

For the decision task, 52% (*SD* = 9%, 95% CI [50%, 55%]) of the items were judged as useful for the environment and hence kept. Mixed-effects logistic regression analysis showed that there was no significant difference in the probability of keeping items between encoding environments (*β* = 0*.*098, *SE* = 0*.*107, Wald *Z* = 0*.*912, *p* = 0*.*362; *M* = 0*.*51, *SD* = 0*.*10, 95% bootstrap CI [0*.*48, 0*.*55]; UW *M* = 0*.*54, *SD* = 0*.*08, 95% bootstrap CI [0*.*51, 0*.*56]). Decision reaction times did not significantly differ between “Useful” (*M* = 793*.*25 ms, *SD* = 271*.*27 ms, 95% bootstrap CI [728*.*18, 860*.*91]) and “Harmful” (*M* = 868*.*58 ms, *SD* = 267*.*64 ms, 95% bootstrap CI [805*.*36, 934*.*33]; *t*(125*.*98) = *−*1*.*582, *p* = 0*.*116) judgments, or between encoding environments (*t*(61*.*25) = 1*.*510, *p* = 0*.*136; MP *M* = 864*.*91 ms, *SD* = 223*.*25 ms, 95% bootstrap CI [787*.*01, 943*.*16]; UW *M* = 775*.*54 ms, *SD* = 249*.*55 ms, 95% bootstrap CI [689*.*70, 864*.*36]).

### Retrieval

**Fig. 2:**
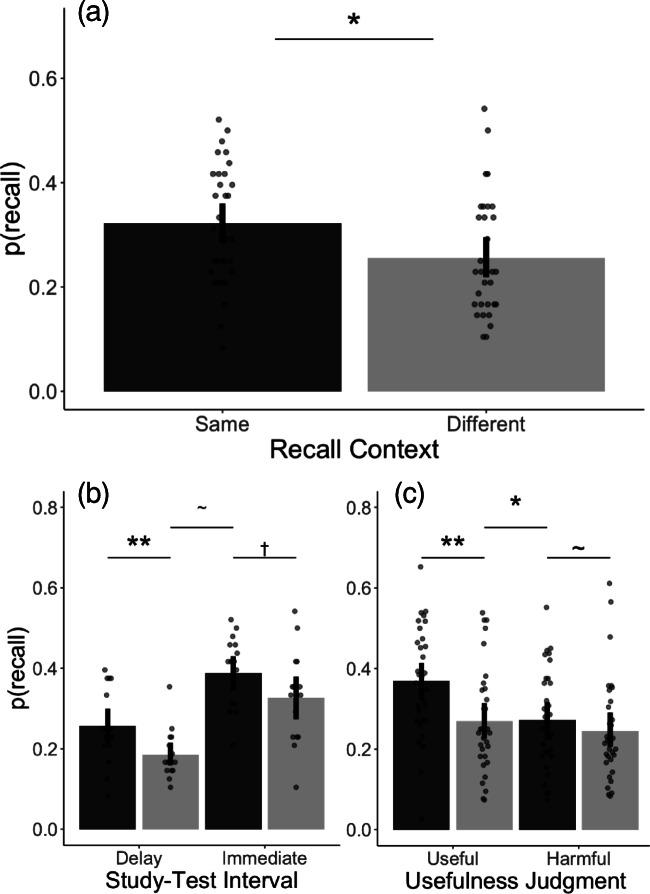
Recall performance. (**a**) The context reinstatement effect. Subjects recalled significantly more words when the recall context was the same as the encoding context (dark gray; N = 32) than when it was different (light gray; N = 32). (**b**) Recall performance as a function of context congruency and study-test interval. The interaction between context congruency and study-test interval was not significant. In the “delay” condition, there was a significant benefit in recalling items in the same context (dark gray; N = 16) compared to the different context (light gray; N = 16). This benefit was not significant in the “immediate” condition (the “same” condition N = 16; the “different” condition N = 16). (**c**) Recall performance as a function of context congruency and usefulness judgment. There was a significant interaction between context congruency and usefulness judgment, where useful items showed a greater benefit from context congruency. Note: Dots indicate individual subjects. Error bars indicate 95% bootstrap confidence intervals. *∗∗ p <* 0*.*01, *∗ p <* 0*.*05, *† p <* 0*.*1, ˜ *n.s.*

Overall accuracy was 0*.*29 (*SD* = 0*.*12, 95% bootstrap CI [0*.*26, 0*.*32]). First, we tested the context reinstatement effect, the benefit in retrieving memory items in the same environment as the encoding environment as opposed to a different environment. Given that a mixed-effects logistic regression model that controlled for a stimulus effect (i.e., word items) as a random effect showed a better fit (*BIC* = 3421*.*5) than a model that did not (*BIC* = 3650*.*0), we used a model with subjects and word items as random effects to predict whether an item is recalled as a function of context congruency; the model also included study-test interval and usefulness judgment as fixed effects. The mixed-effects model showed a significant benefit for the “same” condition (*M* = 0*.*32, *SD* = 0*.*11, 95% bootstrap CI [0*.*28, 0*.*36]) compared to when recall was in a different environment (*M* = 0*.*26, *SD* = 0*.*11, 95% bootstrap CI [0*.*22, 0*.*30]); *β* = 0*.*349, *SE* = 0*.*130, Wald *Z* = 2*.*690, *p <* 0*.*01; Fig. 2a), suggesting that retrieving items is easier when the retrieval environment matches the encoding environment.

To investigate additional factors that affect context reinstatement effect, we tested whether the effect depended on the interval between encoding and retrieval. The interaction between context congruency and interval was not significant (*β* = 0*.*120, *SE* = 0*.*260, Wald *Z* = 0*.*462, *p* = 0*.*644) when tested using the mixed-effects logistic regression model; the effect size (Cohen’s D) for the delay condition was 0.917, and the effect size for the immediate condition was 0.599 (Fig. 2b). There was a main effect of interval where delayed recall performance was significantly worse than immediate recall (*β* = *−*0*.*790, *SE* = 0*.*130, Wald *Z* = *−*6*.*075, *p <* 0*.*001).

To further explore the mechanisms by which context reinstatement affects memory recall, we looked at the relationship between the usefulness judgment and context congruency. The mixed-effects logistic regression model showed an interaction between context congruency and usefulness judgment (*β* = 0*.*384, *SE* = 0*.*179, Wald *Z* = 2*.*137, *p <* 0*.*05), as well as main effects of context congruency (*β* = 0*.*349, *SE* = 0*.*130, Wald *Z* = 2*.*690, *p <* 0*.*01) and usefulness judgment (*β* = 0*.*362, *SE* = 0*.*100, Wald *Z* = 3*.*621, *p <* 0*.*001; Fig. 2c). Planned comparisons showed that the context reinstatement effect was significant among the “useful” items (*β* = 0*.*521, *SE* = 0*.*180, Wald *Z* = 2*.*898, *p <* 0*.*01), but was not significant among the “harmful” items (*β* = 0*.*177, *SE* = 0*.*193, Wald *Z* = 0*.*918, *p* = 0*.*359). These results suggest that reinstating the context preferentially brings back memories for the contextually relevant items, thereby boosting overall memory performance.

## Discussion

In this study, we investigated the environmental context-dependent memory effect in virtual reality, with a design intended to activate distinct bodies of context-specific knowledge. Subjects studied items either underwater (UW) or on Mars planet (MP), under a cover story in which they judged usefulness of items for the given context, and took a surprise free recall test in either the same or different context as the study context. We showed that the items were better recalled when retrieved in the same context as the study context. Importantly, these context-dependent memory effects were only obtained for items that were judged to be useful for survival in the encoding environment.

Circling back to our central motivation, why has it been so difficult to replicate context-dependent memory effects in the laboratory? In the introduction, we hypothesized that the key to obtaining context-dependent memory effects was to integrate items into different schemas (bodies of knowledge) in the two contexts at encoding. The interaction we observed between “usefulness” and context-congruency is consistent with this idea: in our study, the items marked useful at encoding (i.e., items that could be meaningfully integrated into the active schema) were the ones that suffered when the schema active at encoding was not active at recall. Note that this interaction effect cannot be solely explained by deeper encoding of useful items (e.g., survival-related items, Soderstrom & McCabe, 2011) – depth of encoding can explain the main effect of usefulness but not the benefit of reinstating context. Having said this, our study did not directly manipulate schema-integration, so we need to temper our conclusions about the role of schema-integration in driving these effects.

Relating this to the literature more broadly, a potential reason why the seminal Godden and Baddeley (1975) study found such robust effects could be that their subjects were well-familiarized with the sequence of actions of underwater diving as well as those of being on land; consequently, they had “underwater” and “out of the water” schemas that they could use to scaffold knowledge at encoding. Our findings also resonate with prior work showing the importance of integrating items with context (Eich, 1985; Murnane et al., 1999). Eich (1985) showed a larger reinstatement effect in free recall when subjects integrated items into physical contexts (i.e., distinct rooms) by imagining them in the study environment. Similarly, Murnane et al. (1999) found larger context-change effects on recognition sensitivity when items could be integrated into a familiar situation (e.g., when words were shown on a picture of a blackboard in a classroom) versus when they could not (e.g., when the context was defined by combinations of word color, background color, and location). Our usefulness-judgment results extend this idea by showing that simply having a meaningful context (i.e., one that activates an existing schema) is not sufficient to yield context-dependent memory; rather, subjects have to actually succeed in integrating the item into the encoding context (i.e., the item has to be judged to be “useful” in that context) to get an effect of context congruency for that item at recall.

Our study used VR in order to provide both perceptually and semantically rich experiences. The immersive nature of VR makes it a potentially useful tool for studying context-dependent memory (Dunsmoor et al., 2014; Reggente et al., 2018). However, the use of VR does not guarantee a context-dependent memory effect. For instance, a recent study that also used VR did not find the context-reinstatement effect (Wälti et al., 2019). In their study, Wälti and colleagues asked subjects to remember a list of words presented on a background image, following the study by Isarida and Isarida (2007) where contexts were manipulated using the background color for studied words. On each trial, a word was presented on a context image (i.e., a background color, a landscape picture, a virtual background, or background flickering) for 3 s, and the context was pseudorandomly selected on each trial such that the same context did not appear for more than three words in a row.

While we also used VR, our study significantly differs from the Wälti et al. (2019) study. Wälti and colleagues used VR to present backgrounds and did not allow direct interaction with these backgrounds; by contrast, we used highly interactive virtual environments to encourage activation of distinct schemas in the two environments. Moreover, our study used a surprise memory test to prevent participants from using other strategies that could potentially suppress processing of context information. Lastly, the frequency of context switches was higher in the Wälti study – there were at least seven context shifts during encoding. This context manipulation may have been ineffective because the rate of context switch was too rapid to match the human prior for a context change. For instance, it is unlikely that the room where we read emails changes as quickly as the switch of tabs on a browser or apps on a phone. In other words, it may be that contexts shift at hierarchically different timescales (Collins & Frank, 2013; Kurby & Zacks, 2008; Zacks et al., 2001). If the two alternating contexts form a higher-level context in which there is an alternation between the two background images with certain transition probabilities, then what is designated as the “change” or “different context” condition may not actually involve a context change (since subjects continue with same higher-level mental context active in mind). Another implication of the idea that changes in the internal context representation drive memory effects (more so than changes in sensory input) is that asking participants to mentally reinstate the encoding context should reduce the size of the context-dependent memory effect as in Smith (1979); this is a promising direction for future work.

Lastly, in addition to the factors outlined above, we manipulated study-test delay: There were two delay conditions, one in which subjects were tested immediately after encoding and another after a 1-day delay. Our data show that the benefit of context reinstatement for the 1-day delay was numerically (but not significantly) larger than that of immediate recall. Increasing the delay might boost context-dependent memory effects by boosting the extent to which participants rely on hippocampally-mediated episodic memory (Chen et al., 2016) instead of active maintenance of items in working memory (for a review, see Richmond & Zacks, 2017). Hippocampal codes are known to be highly context-dependent (Eichenbaum, 2004), so it follows that anything that increases reliance on the hippocampus should increase context-dependence. Additionally, studies have found that schema-consistent information undergoes accelerated consolidation into neocortex, mediated by interactions between hippocampus and medial prefrontal cortex (Gilboa & Marlatte, 2017; Wang & Morris, 2010); an extra day of consolidation could boost context-dependent memory effects by fostering additional integration of schema-consistent items with their context. Future studies can use neuroimaging to test the role of hippocampal engagement and hippocampal-neocortical interactions in driving the context-dependent recall effects observed here.

In summary, we showed a context reinstatement effect using environments that were designed to activate pre-existing schemas (i.e., underwater and outer-space planet environments), and schema-consistent items were most likely to show context-dependent memory effects. These results suggest that integration of items into active schemas plays a key role in driving context-dependent recall effects. However, identifying the exact features of our paradigm that were necessary and sufficient for obtaining these effects will require additional research.
